# Activation of the pattern recognition receptor NOD1 in periodontitis impairs the osteogenic capacity of human periodontal ligament stem cells via p38/MAPK signalling

**DOI:** 10.1111/cpr.13330

**Published:** 2022-08-31

**Authors:** Yuying He, Zuping Wu, Sirui Chen, Jiahe Wang, Li Zhu, Jing Xie, Chenchen Zhou, Shujuan Zou

**Affiliations:** ^1^ State Key Laboratory of Oral Diseases, West China Hospital of Stomatology Sichuan University Chengdu China; ^2^ National Clinical Research Center for Oral Diseases, West China Hospital of Stomatology Sichuan University Chengdu China

## Abstract

**Objectives:**

Nucleotide oligomerization domain receptor 1 (NOD1) mediates host recognition of pathogenic bacteria in periodontium. However, the specific role of NOD1 in regulating osteogenesis is unclear. Therefore, this study focused on the activation status of NOD1 in periodontitis and its effect on the osteogenic capacity of human periodontal ligament stem cells (hPDLSCs) as well as the underlying mechanism.

**Methods:**

Histological staining and Western blot were utilized to assess NOD1 expression in the periodontium of people with or without periodontitis. HPDLSCs were cultured under NOD1 agonist or antagonist treatment. Q‐PCR and Western blot were employed to assess the expression of osteogenic marker genes and proteins. Alizarin red staining and alkaline phosphatase staining were used to determine the osteogenic capability of hPDLSCs. The activation of downstream signalling was determined and specific inhibitors were utilized to confirm the signalling pathway in NOD1‐regulated osteogenesis.

**Results:**

NOD1 expression is significantly elevated in periodontitis. With NOD1 activated by particular agonist tri‐DAP, the osteogenic potential of hPDLSCs was impaired. NOD1 antagonist co‐incubation partially restored the decreased osteogenesis in hPDLSCs. P38/MAPK was phosphorylated in tri‐DAP‐induced NOD1 activation. The inhibitor of p38 rescued the suppression of osteogenesis induced by tri‐DAP in hPDLSCs.

**Conclusions:**

Our study revealed the expression status of NOD1 in periodontitis. Its activation greatly decreased the osteogenic capacity of hPDLSCs which was mediated by the phosphorylation of p38 downstream signalling.

## Graphical abstract

Nucleotide oligomerization domain receptor 1 (NOD1) is substantially expressed in periodontium of individuals with chronic periodontitis. Abnormal activation of NOD1 may result in alveolar bone loss by impairing the osteogenic potential of human periodontal ligament stem cells, which provides a potential target in periodontium regeneration.

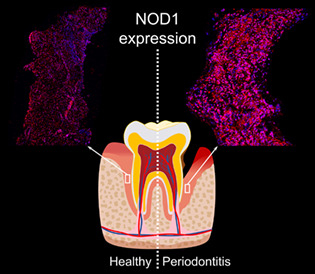

## 1 INTRODUCTION

Periodontitis is a chronic dental infection initiated by a polymicrobial community and characterized by periodontium degeneration and destruction.^1^, ^2^, ^3^ The rise of a pathogenic community means the presence of diverse high‐level bacterial components that interfere with the host and result in subsequent alveolar bone loss.^4^ The virulence factors that have been characterized include adhesins, proteolytic enzymes, cognate receptors, and proinflammatory surface ligands/structures.^5^ Recent studies suggest these adverse molecules may induce unresolving local alveolar bone destruction by decreasing the regeneration capacity of periodontal connective tissues.^6^, ^7^, ^8^, ^9^

As a major cell source with the osteogenic potential to regenerate alveolar bone, human periodontal ligament stem cells (hPDLSCs) are amongst the primary host cells that directly interact with invading microbe in the periodontium. Bone regeneration capacity of hPDLSCs may be impacted by elevated oral bacterial burden during periodontal disease.^10^, ^11^ Being an essential part of this microbe–host interaction, hPDLSCs can recognize periodontal pathogens through the pattern‐recognition receptors (PRRs) constitutively expressed in the cells.^12^ These virulence factors that can be recognized by PRRs are pathogen‐associated molecular patterns (PAMPs), which are constitutive or secreted molecules that are structurally conserved in most microbes.^13^ Nucleotide oligomerization domain receptor 1 (NOD1) is a cytosolic PRR from the NLRs family that recognize the substructure of bacterial peptidoglycans (PGN), γ‐d‐glutamyl‐meso‐diaminopimelic acid (iE‐DAP), which can be released by most periodontal pathogen.^14^, ^15^, ^16^, ^17^ Once sensing iE‐DAP, NOD1 induces CARD‐CARD interaction and activates NF‐κB and MAPK signalling for triggering inflammatory responses.^18^, ^19^

Current studies regarding the role of NOD1 in periodontitis bone loss remain ambiguous. Activation of NOD1 can induce potent secretion of chemokines and other proinflammatory molecules, which mediate tissue and bone destruction.^20^, ^21^ Jiao et al. discovered that NOD1 knockout mice were less susceptible to periodontitis, revealing that NOD1 is essential for the recruitment of neutrophils and osteoclasts.^22^ However, contrary results were shown in another mice periodontitis model which suggests a bone‐sparing role for NOD1.^23^ Multiple oral bacteria that have been considered to have a strong association with periodontitis can modulate host behaviour through the NOD1 receptor. *Porphyromonas gingivalis* induces intracellular adhesion molecule production which is correlated to the severity of periodontitis and this process can be mediated by NOD1 in periodontal fibroblasts.^24^ *Actinobacillus actinomycetemcomitans* generates leukotoxins that are toxic to host leukocytes and provides immunostimulation via NOD1.^22^, ^25^ *Fusobacterium nucleatum* stimulates neutrophils and generates neutrophil extracellular traps, which are critical in the fight against pathogenic microbes through the upregulation of NOD1.^26^ These immune responses are crucial for phagocytic immune cells recruitment and sequential inflammatory bone loss.^4^ The above results provide an insight that signalling via PGN‐NOD1 may connect bacteria with alveolar bone loss.

NOD1 has been affirmed as an important cytosolic sensor for microbial PGNs in the oral cavity.^26^, ^27^, ^28^, ^29^ It is functionally expressed in human periodontal ligament cells mediating host recognition of PGNs containing iE‐DAP.^19^, ^27^ To date, there is no study characterizing whether NOD1 activation directly impacts the osteogenic differentiation process in hPDLSCs. In this study, we detect considerably elevated levels of NOD1 expression in periodontal tissues in periodontitis. We use tri‐DAP as an agonist to mimic the heavy pathogen burden in the periodontium and successfully activate NOD1 in‐vitro. We conclude that NOD1 activation can decrease the osteogenic capacity of hPDLSCs via the p38 MAPK pathway. Our findings indicate a novel role of NOD1 in microbe‐affected osteogenesis in periodontitis and present a possible target for clinical treatment.

## 2 METHOD AND MATERIALS

### 2.1 Samples collection

Periodontal ligament (PDL) tissues were obtained from five adult patients with periodontitis and five healthy adult patients receiving orthodontic treatment at West China Hospital of Stomatology. The procedures met the requirement of ethical principles (WCHSIRB‐D‐2021‐537, Sichuan University, Chengdu, China). Healthy periodontium was defined as periodontal probing depths less than 2 mm with no more than 15% bleeding on probing. Healthy periodontal ligament tissue was obtained from teeth extracted in orthodontic cases or wisdom teeth without inflammation. Periodontitis was defined when there is at least one site of probing depth exceeding 4 mm. And the periodontal ligament was retrieved from those hopeless teeth that had to be excised based on the treatment plan. After obtaining the teeth from dental procedures, we gently scraped off the remaining periodontal ligament tissues from the surface of the teeth with a scalpel. These samples from clinical participants were employed for protein level analysis, Hematoxylin and Eosin (H&E) staining, Immunohistochemistry (IHC) staining, and Immunofluorescence (IF) staining.

### 2.2 HPDLSCs isolation and cultivation

HPDLSCs were separated from the periodontal tissues obtained from healthy third molars or premolars. Teeth extraction was conducted on patients with orthodontic or dental therapeutic reasons following informed consent at West China Hospital of Stomatology. Following the extraction, PDL tissues were carefully scraped and processed with collagenase type I (Gibco) for 20 min. Tissue pieces were then cultured in a humidified environment with 5% CO_2_ at 37°C in a growth medium (α‐MEM, Sigma–Aldrich) containing 10% foetal bovine serum (Invitrogen) and 1% penicillin–streptomycin (Hyclone). HPDLSCs in passages 2–6 were used in our experiments.

### 2.3 Cell treatment

To activate or inhibit NOD1 and its downstream signalling ways, we referred to the relevant literature^27^, ^28^ and treated hPDLSCs with various doses of ligands as follows according to the pre‐experimental results (Figure S3): l‐alanyl‐γ‐d‐glutamyl‐meso‐diaminopimelic acid (tri‐DAP, comprises iE‐DAP and a L‐Ala residue, henceforth termed as DAP; tlrl‐tdap, Invivogen; 1–10 μg/ml) was used as NOD1 agonist; ML130 (4354/10, Tocris Bioscience; 20 μM); SB203580 (#5633, Cell Signaling Technology; 10 μM) was utilized as NOD1 and p38 inhibitor respectively.

### 2.4 Osteogenic differentiation

HPDLSCs were induced in an α‐MEM medium containing 5% foetal bovine serum, 1% penicillin–streptomycin, 0.02 μM dexamethasone (Sigma–Aldrich), 50 μg/ml l‐ascorbic acid (J&K), and 8 mM glycerophosphate (Sigma–Aldrich). After 3 and 7 days of culture, an alkaline phosphatase (ALP) assay was used to detect osteogenic differentiation. After 14 days of culture, alizarin red staining was used to evaluate calcified nodule formation in the extracellular matrix. RNA and protein expressions of osteogenesis‐related markers were detected by qRT‐PCR and Western blot on day 3 and day 7, respectively.

### 2.5 Alkaline phosphatase (ALP) staining

After 3 or 7 days of culture, cells were first rinsed in phosphate‐buffered saline (PBS) two to three times before being fixed in 4% paraformaldehyde (PFA) for 15 min. Staining was conducted with BCIP/NBT dye solution at room temperature, shielded from light, for 15–30 min. Images were observed with a light microscope. The BCIP/NBT Alkaline Phosphatase Color Development Kit (Beyotime) was applied in this assay.

### 2.6 Alizarin red staining

After 10–14 days of culture, hPDLSCs were washed with PBS, fixed in 4% PFA for 15 min, and stained for 30–60 min with a 1% Alizarin Red Staining Solution (Solabio) under gentle agitation. After staining, gently wash off the residual dye solution with distilled water. A light microscope was applied to observe and photograph to stained calcified nodules.

### 2.7 Protein extraction and Western blot

Protein samples were extracted employing radioimmunoprecipitation assay lysis buffer (Biosharp) containing the protease inhibitor cocktail (Sigma–Aldrich) as directed by the manufacturer. After protein concentration was measured, proteins were first separated on a polyacrylamide gel and then deposited into polyvinylidene difluoride (PVDF) blots (Millipore, Billerica). Following the transfer, the blotting membranes were incubated with primary antibodies targeting NOD1 (Bioss Antibodies); phosphorylated P38, P38, phosphorylated ERK, ERK, phosphorylated JNK, JNK, Collagen I, Osterix, ALP, and Runx2 (ZEN BIO) were used in this study. Next, the membranes were treated with secondary antibodies for 2 h at room temperature. Then, protein bands were identified by enhanced chemiluminescence (ECL) reagents (Santa Cruz) treatment and exposure of x‐ray film. Protein bands were quantified using the Image J software.

### 2.8 Real‐time quantitative reverse transcription PCR (qRT‐PCR)

Total RNA was isolated from hPDLSCs using an RNApure total RNA fast isolation Kit (Bio Teke) and was quantitated with the Nanodrop spectrophotometer (Thermo Fisher Scientific). Then, RNA samples were reverse‐transcribed into cDNA with RevertAid First Strand cDNA Synthesis Kit (Thermo Fisher Scientific). Real‐time PCR reactions were performed in a Bio‐Rad system following the manufacturer's instructions. The cycle threshold value and the 2^−ΔΔCt^ method were used in calculating the osteogenesis‐related gene expression using GAPDH as a control gene. Primer sequences of genes detected in this study are listed in Table S1.

### 2.9 Immunohistochemistry

The periodontal tissue samples were fixed in 4% PFA for 12 h. After dehydration, the samples were embedded into paraffin blocks and cut into sections at 4‐μm thickness using a microtome (Leica RM2235). Then, sections were transferred onto adhesive slides and were deparaffinized with xylene and rehydrated with descending concentrations of ethanol. The antigen retrieval step was performed by incubating the slides with antigenic repair fluid at 95°C for 10 min. Following cooling down, slides were rinsed in PBS solution. 3% H_2_O_2_ and 5% bovine serum albumin (BSA) were used to block the endogenous peroxidase activity. Incubation with the primary antibodies was carried out at 4°C overnight using rabbit anti‐CARD4 (Bioss Antibodies). The slides were then rinsed in PBS solution and treated with secondary antibody for 2 h. Next, the location of NOD1 was visualized using the DAB solution kit (Sigma–Aldrich). Finally, the slides were counterstained with haematoxylin and eosin, and observed with a light microscopy (Olympus).

### 2.10 Immunofluorescence

Protein expression and distribution in hPDLSCs and periodontal tissues were investigated using immunofluorescence. Cell samples were rinsed in PBS and fixed with 4% PFA after 2 h of DAP treatment. Next, permeabilized the membrane of cells with 0.5% Triton X‐100 and blocked the samples in PBS containing 1% BSA for 1 h. Following incubation overnight at 4°C with the primary antibody Phospho‐p38 (ZEN BIO), cells were incubated with the fluorescein‐conjugated anti‐rabbit IgG AlexaFluor®647 (Abcam) for 2 h. The cytoskeleton was stained with phalloidin (Thermo Fisher Scientific) and the nuclei was stained with 4′, 6‐diamidino‐2‐phenylindole (DAPI; Sigma). The preparation of tissue slices was identical to that used for immunohistochemistry. Rabbit anti‐CARD4 antibodies (Bioss Antibodies) were used as primary antibodies. Anti‐rabbit IgG (Alexa Fluor® 647, Abcam) was applied as secondary antibodies. Nuclear staining was performed with DAPI (Sigma). The fluorescence staining of cells and tissues was examined using CLSM (FV3000, Olympus, Japan).

### 2.11 Statistical analysis

We utilized *t*‐test or one‐way ANOVA for multiple continuous variables comparisons, and comparative results within each experiment were made against the control or baseline. The results are expressed as the mean ± standard error (SEM) of at least three independent experiments. Analysis and graph preparation was accomplished using the Prism® GraphPad version 9.

## 3 RESULTS

### 3.1 NOD1 is highly expressed in patients with chronic periodontitis

**FIGURE 1:**
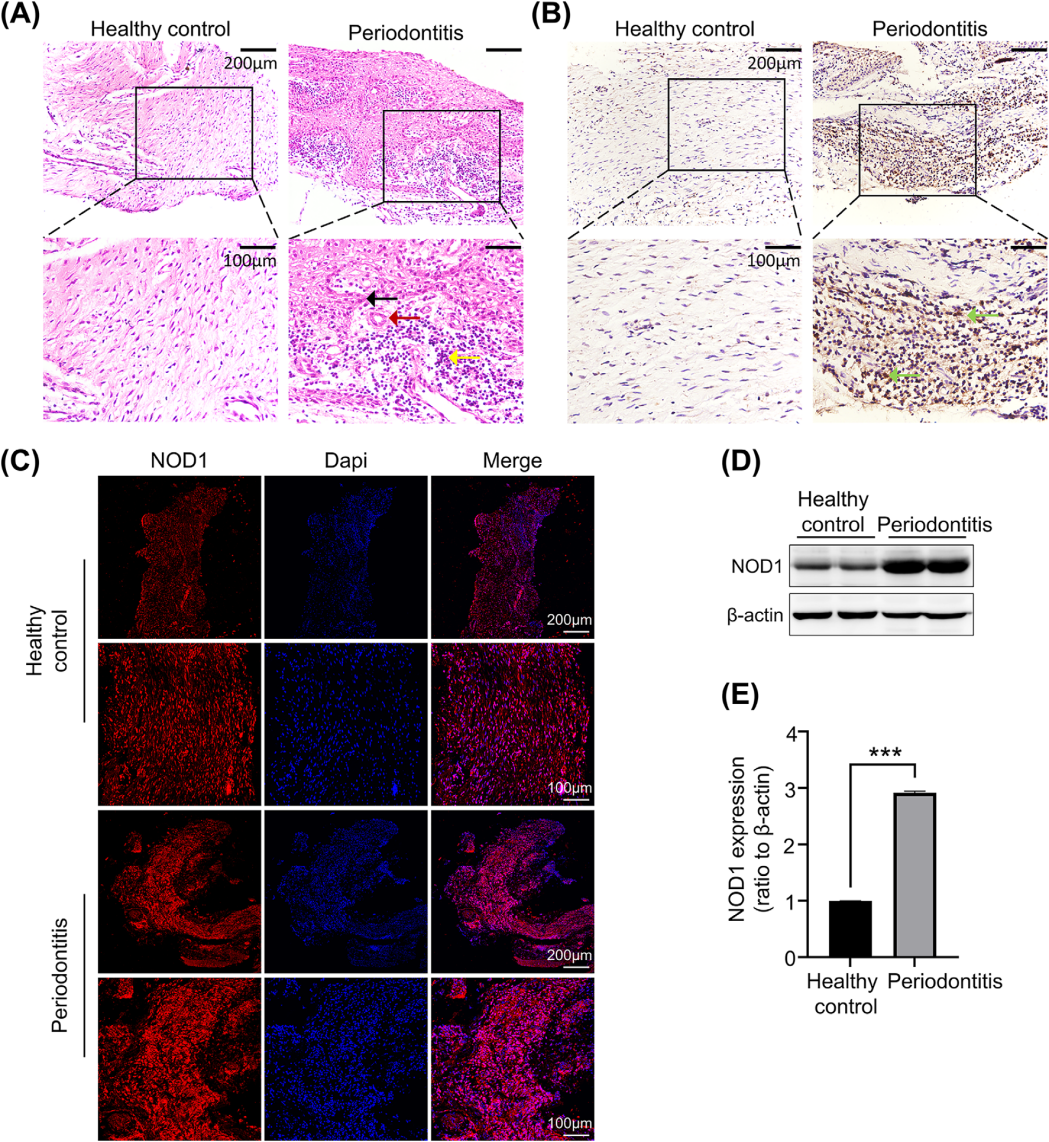
NOD1 is strongly expressed in the periodontium of patients with periodontitis. (A) Representative H&E staining images of periodontal tissue harvested from healthy people and patients with periodontitis. Arrows presented the elongated epithelial ridges, the proliferation of blood vessels, and accumulation of inflammatory cells in periodontitis periodontium respectively. (B) Representative IHC staining images demonstrating the substantial alterations in NOD1 in the subepithelial connective tissue in periodontal tissue. (C) Immunofluorescence staining showing the presence and distribution of NOD1 in the periodontium. (D) Western blot showing the protein level of NOD1 expression in the periodontium. Protein samples are collected from five periodontitis adults and five healthy adults. Histograms showed the quantitative analysis of NOD1 expression. Three independent experiments (*n* = 3) were used in the statistical analysis (***p < 0.001).

To determine the level of NOD1 expression in periodontitis. Periodontal tissues were harvested from the healthy teeth pulled out for orthodontics treatment and the loose teeth pulled out due to chronic periodontitis. The samples collection process was shown in Figure S2. H&E staining showed that under local periodontal inflammation, periodontal ligament exhibited thickened and elongated epithelial ridges with abundant inflammatory cell accumulation within the lamina propria compared to the healthy control (Figure 1A). Immunohistochemistry (IHC) staining and Immunofluorescence (IF) staining were carried out to confirm the presence and distribution of NOD1 in periodontitis. The result showed a noteworthy increase of NOD1 in the subepithelial connective tissue in the inflamed periodontal tissue, indicating that NOD1 may participate in the progression of periodontal diseases (Figure 1B,C). Moreover, proteins were extracted from two groups of periodontal tissues, and subsequent Western blot detected significantly higher NOD1 expression in patients with chronic periodontitis (Figure 1D).

These results indicated that NOD1 was remarkably activated in periodontal tissue when host inflammation occurred, which prompted NOD1 activation may play an important role in alveolar bone loss in chronic periodontitis.

### 3.2 DAP, a NOD1 agonist, inhibited the osteogenic differentiation of hPDLSCs

**FIGURE 2:**
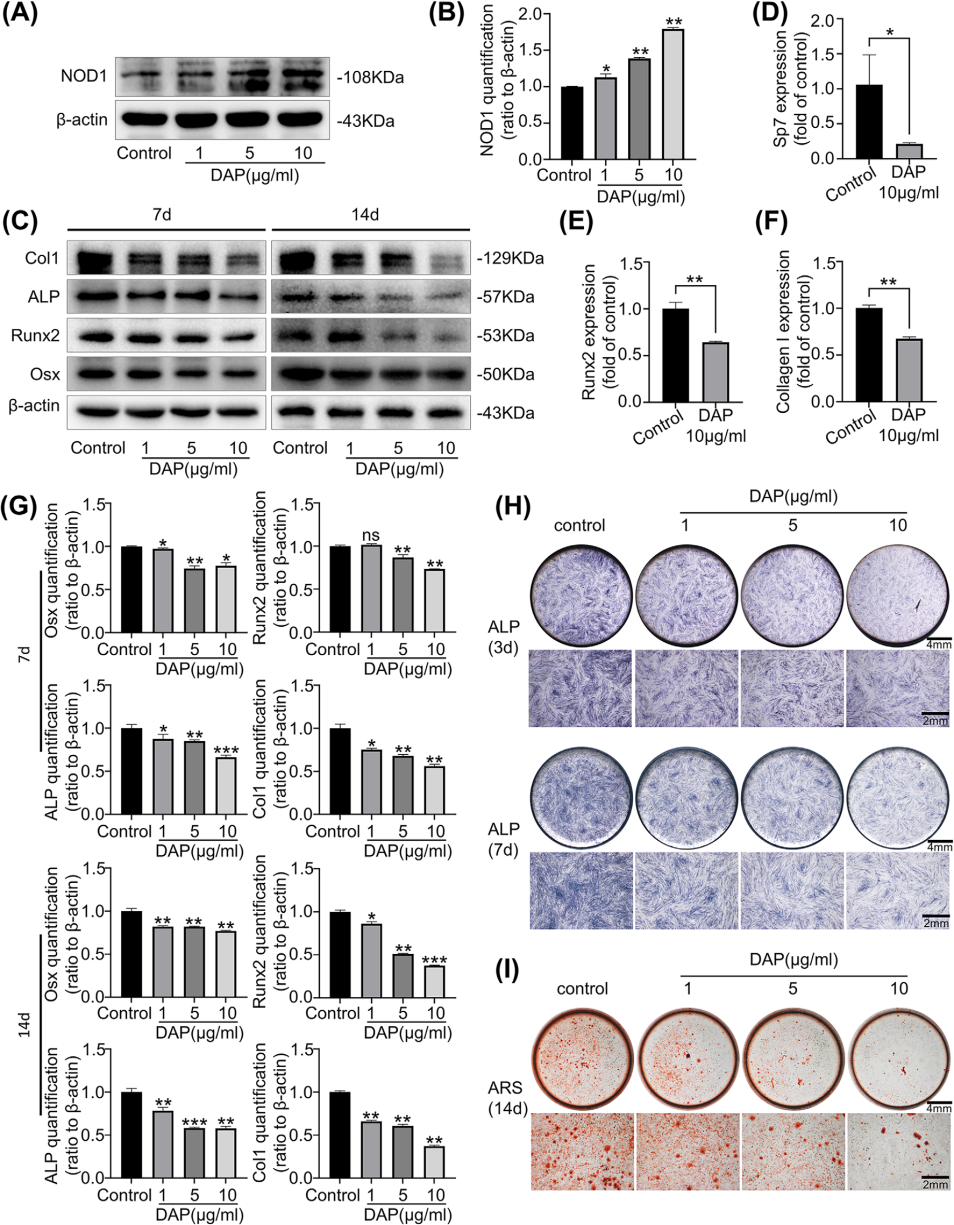
DAP‐induced NOD1 activation inhibited the osteogenic capacity of hPDLSCs. (A) Western bolt showing NOD1 expression in hPDLSCs stimulated by DAP from 1 μg/ml to 10 μg/ml for 2 h. Three separate tests (*n* = 3) yielded similar results. (B) Quantification showing the protein changes of NOD1 at 2 h treatment of DAP. NOD1 expression was significantly increased at DAP (10 μg/ml) incubation (*n* = 3) (*p < 0.05, **p < 0.01). (C) Western blot demonstrating the expression of osteogenesis‐related proteins, including Col1, ALP, Runx2, and Osx in hPDLSCs treated with different doses of DAP after 7 and 14 days of osteogenic differentiation. Three separate tests (*n* = 3) yielded similar results. (D–F) RT‐qPCR data demonstrating the level of osteogenesis‐related gene expression, including SP7, Runx2, and Collagen I in hPDLSCs under DAP (10 μg/ml) treatment after 7 days of osteogenic differentiation. Three independent experiments (*n* = 3) were used in the statistical analysis (*p < 0.05, **p < 0.01). (G) Quantification showing the protein changes of osteogenic markers presented in C (*n* = 3) (*p < 0.05, **p < 0.01, ***p < 0.001). (H, I) HPDLSCs were cultured with DAP at doses ranging from 0 to 10 μg/ml in osteogenic media. ALP staining showed the decreased ALP activity of hPDLSCs at 3 and 7 days. Alizarin red staining showed the reduced calcified nodule formation in hPDLSCs at 14 days.

First, based on the previous study of our research group,^29^ we cultured hPDLSCs expressing the mesenchymal stem cell markers CD90 and CD146 and characterized its multidirectional differentiation potential (Figure S1). To evaluate whether the activation of NOD1 affects the osteogenic potential of hPDLSCs, hPDLSCs were stimulated with DAP, a highly selective NOD1 agonist. The in‐vitro effect of NOD1 activation with DAP was assessed by Western blot. DAP treatment profoundly elevated the expression of NOD1 in hPDLSCs after 60 min of incubation in a dose‐dependent manner (Figure 2A,B).

ALP staining showed DAP treatment significantly reduced ALP activity after 3 and 7 days of osteogenic differentiation in a dose‐dependent pattern (Figure 2H). Alizarin red staining presented a suppressed mineralized nodule formation on day 14 with DAP treatment (Figure 2I). Osteogenic markers (e.g., RUNX2, Collagen I, and SP7) gene mRNA expression was also inhibited by DAP stimulation. And the maximal down‐regulation was achieved at 10 μg/ml of DAP (Figure 2D–F). Further, Western blotting verified that DAP (10 μg/ml) incubation decreased the protein expression of Col1, ALP, Runx2, and Osx in hPDLSCs on days 7 and 14, respectively (Figure 2C,G).

These findings imply that NOD1 activation impairs the osteogenic capacity of hPDLSCs.

### 3.3 Inhibition of NOD1 rescues the suppression of osteogenic capacity mediated by DAP

**FIGURE 3:**
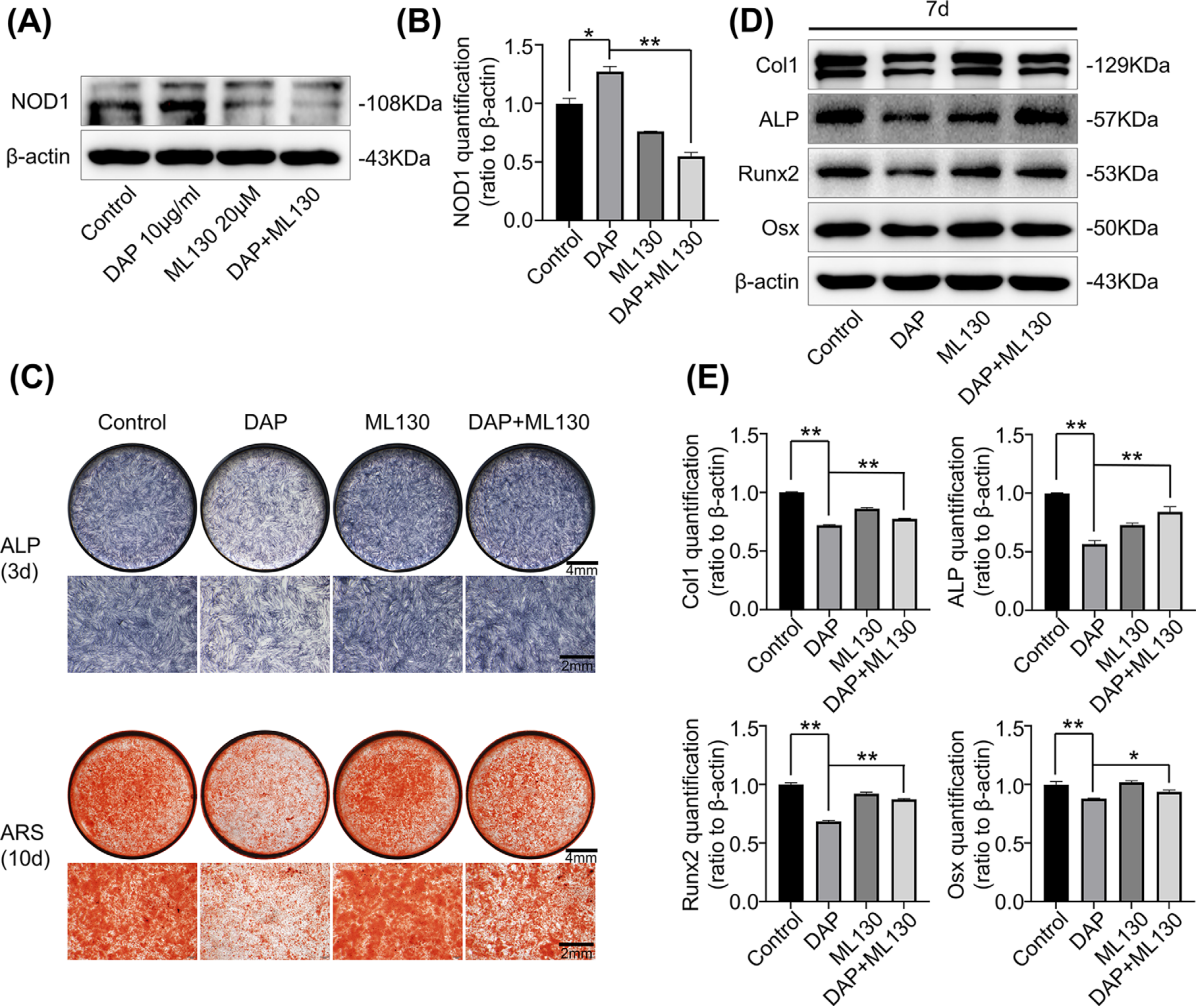
Inhibition of NOD1 partially rescued the DAP‐impaired osteogenesis in hPDLSCs. (A) Western bolt showing NOD1 expression in hPDLSCs with DAP (10 μg/ml), ML130 (20 μM), DAP (10 μg/ml) and ML130 (20 μM) co‐incubation treatment for 2 h respectively. Three separate tests (*n* = 3) yielded similar results. (B) Quantitative analysis showing the protein alterations of NOD1 in hPDLSCs (*n* = 3) (*p < 0.05, **p < 0.01). The results implied that the activation of NOD1 with DAP (10 μg/ml) incubation was inhibited by ML130 (20 μM). (C) ALP staining and alizarin red staining images showing the ALP activity and calcified nodule formation of hPDLSCs after 3 and 10 days of osteogenic differentiation with DAP (10 μg/ml) and ML130 (20 μM). (D) Western blot demonstrating the expression of osteogenesis‐related proteins, including Col1, ALP, Runx2, and Osx in hPDLSCs under DAP (10 μg/ml) and ML130 (20 μM) incubation for 7 days. Three separate tests (*n* = 3) yielded similar results. (E) Quantification showing the protein changes of osteogenic markers presented in (D). The decreased osteogenic marker levels induced by DAP (10 μg/ml) were abolished by ML130 (20 μM) co‐incubation (*n* = 3) (*p < 0.05, **p < 0.01).

To verify the significant involvement of NOD1 in DAP‐affected osteogenic differentiation in hPDLSCs, ML130, a chemical synthesized antagonist of NOD1 was employed to block NOD1. The in‐vitro impact of DAP treatment upon NOD1 activation was suppressed by co‐incubation with ML130 at 20 μM (Figure 3A,B). The ALP activity and calcified nodule formation inhibited by DAP incubation were reverted by the addition of ML130 (Figure 3C). Western blot also indicated that the decrease of osteogenic capacity in the DAP‐induced hPDLSCs was abrogated with ML130 co‐incubation (Figure 3D,E).

On the basis of these findings, we infer that inhibiting NOD1 reverses DAP‐impaired osteogenic differentiation in hPDLSCs, suggesting that NOD1 activation interferes with the osteogenic process in hPDLSCs.

### 3.4 NOD1 activation induces P38 MAPK downstream signalling in hPDLSCs

A previous study reported that NOD1 activation in human periodontal ligament cells (hPDLCs) could trigger MAPK signalling in inflammatory response.^27^ To further delineate the mechanism of DAP‐NOD1 suppressing osteogenic differentiation of hPDLSCs, we start to focus on the phosphorylation of MAPKs with DAP treatment.

**FIGURE 4:**
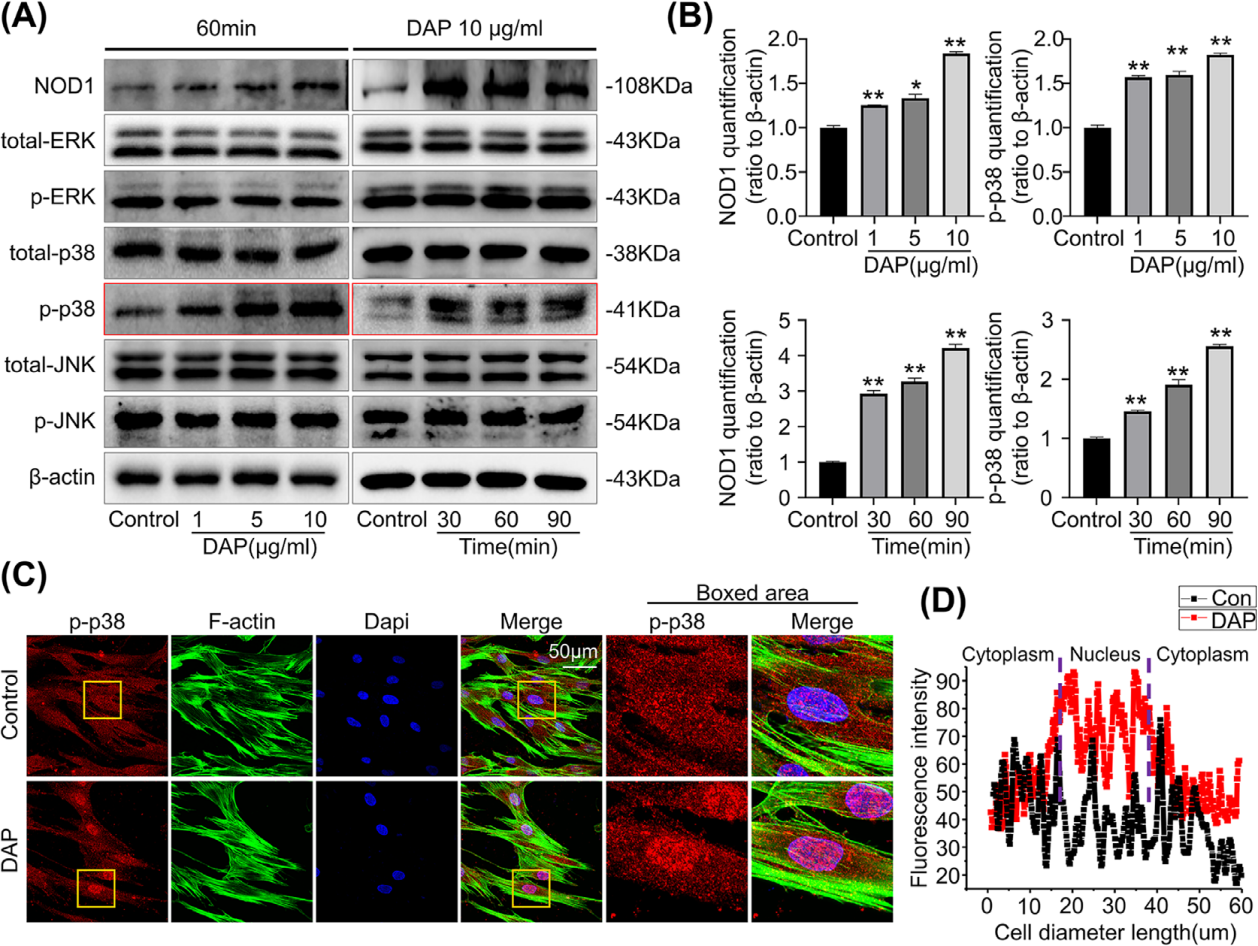
DAP induced NOD1 activation and increased phosphorylation of p38. (A) Representative Western blot results demonstrating the expression of NOD1 and the phosphorylated MAPK molecules in hPDLSCs stimulated with DAP (0–10 μg/ml) for 60 min and DAP (10 μg/ml) for 0–90 min respectively. B. Quantitative analysis indicating the changes in NOD1 and phosphorylated p38 showing that NOD1 activation induces the phosphorylation of p38. Three separate experiments (n = 3) were used in the statistical analysis (*p < 0.05, **p < 0.01). (C) Representative IF staining showing the changes of phosphorylated p38 (red) in hPDLSCs after 2 h of DAP incubation. The cytoskeleton was stained green by phalloidin reagent and the nucleus was stained blue by DAPI. (D) Fluorescence optical density (OD) assay showing the nucleus distribution of phosphorylated p38 in DAP‐treated hPDLSCs presented in (C).

When NOD1 was activated by raising doses of DAP to 10 g/ml and increasing incubation duration to 90 min, we observed a significant increase in p38 phosphorylation (Figure 4A,B). While such dose‐ and time‐dependent activation were not observed on other MAPK signalling pathway components, including JNK, pJNK, ERK, and p‐ERK. IF staining also showed an evident nucleus accumulation of phosphorylated p38 in hPDLSCs with 90 min of DAP (10 μg/ml) incubation (Figure 4C).

Therefore, we suggest that NOD1 activation in hPDLSCs was mediated by the subsequent phosphorylation of p38.

### 3.5 Inhibitor of P38 MAPK restores the DAP‐impaired osteogenic capacity of hPDLSCs

**FIGURE 5:**
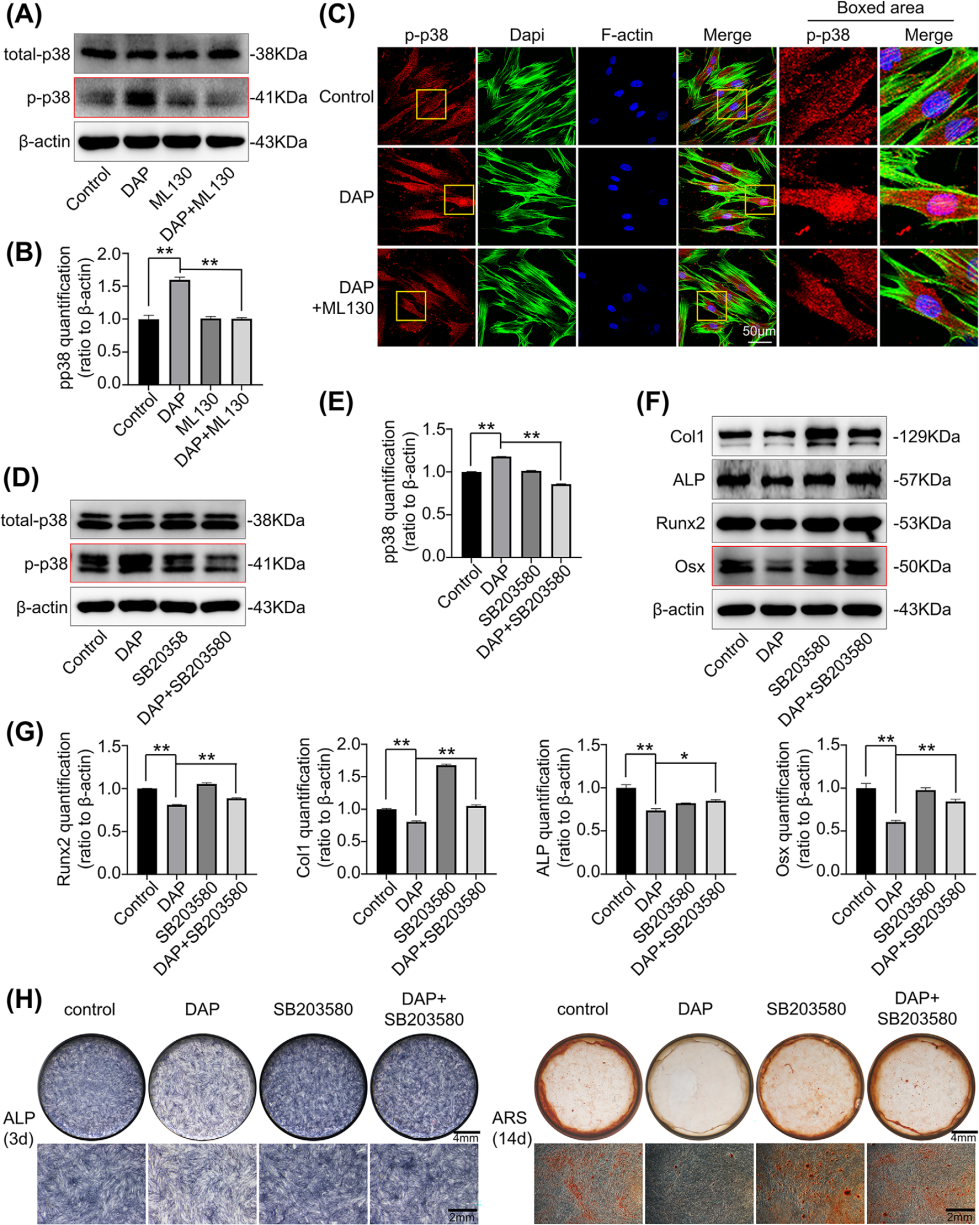
P38 inhibitor restored the DAP‐decreased osteogenic capacity of hPDLSCs. (A, B) Western blot results presenting the expression of phosphorylated p38 in hPDLSCs under DAP (10 μg/ml) and ML130 (20 μM) treatment for 90 min. Quantitative analysis in B affirmed the protein changes shown in (A), which indicated ML130 treatment successfully abolished DAP‐induced upregulation of p38 phosphorylation. Three independent experiments (*n* = 3) were used in the statistical analysis (*p < 0.05, **p < 0.01). (C) Immunofluorescence staining showing the distribution of phosphorylated p38 in hPDLSCs with DAP (10 μg/ml) and ML130 (20 μM) co‐incubation for 2 h. (D, E) Western blot results demonstrating the protein level of phosphorylated p38 in hPDLSCs stimulated by DAP (10 μg/ml) and SB203580 (10 μM) for 90 min. Three separate tests (*n* = 3) were used in quantification (E) confirming the protein changes shown in (C) (*p < 0.05, **p < 0.01). (F) Western blot results demonstrating the protein expression of osteogenic markers in hPDLSCs with DAP (10 μg/ml) and SB203580 (10 μM) incubation for 7 days. Three separate tests (*n* = 3) yielded similar results. (G) Quantitative analysis confirmed the protein changes presented in (F). The decrease of osteogenic marker levels induced by DAP (10 μg/ml) were abrogated by SB203580 (10 μM) co‐incubation (*p < 0.05, **p < 0.01) (*n* = 3). (H) ALP staining and alizarin red staining showing the ALP activity and mineralized nodule formation of hPDLSCs after 3 and 14 days of osteogenic differentiation with DAP (10 μg/ml) and SB203580 (10 μM).

To corroborate p38 MAPK as the important downstream signalling in NOD1 activation, the quantity of phosphorylated p38 was measured with the addition of ML130 and the p38 MAPK selective inhibitor SB203580. The upgraded p38 phosphorylation in hPDLSCs treated with DAP was blocked by ML130 co‐incubation (Figure 5A,B). Consistent with this result, IF staining validated that the p38 phosphorylation level was successfully down‐regulated by ML130 (Figure 5C). These results affirmed that NOD1 activation in hPDLSCs was mediated by the phosphorylation of p38.

Then, we applied SB203580 and evaluated its effect on the osteogenesis process in hPDLSCs, to confirm the observed biochemical alterations in p38 phosphorylation corresponded to the osteogenic phenotypes observed in DAP‐induced hPDLSCs. The phosphorylation of p38 was no longer upregulated by DAP after SB203580 treatment (Figure 5D,E). We performed ALP staining and alizarin red staining on hPDLSCs incubated with DAP and SB203580. Consistent with the previous tests, DAP treatment reduced the ALP activity (Day 3) and the formation of mineralized nodules (Day 14). SB203580 co‐incubation restored the impaired ALP activity and mineralized nodule formation in hPDLSCs (Figure 5H). Western blotting identified similar results, presenting a recovered osteogenic marker protein level trend after using SB203580 (Figure 5F,G).

These findings further demonstrated that p38 phosphorylation plays an essential role in the NOD1‐regulated osteogenic differentiation of hPDLSCs.

## 4 DISCUSSION

Mounting evidence has proved the strong connection between oral microbe and periodontitis. Recent studies suggested that host damage and immunostimulation induced by bacteria act synergistically in periodontitis.^30^ Thus, we need to seek out the potential mechanism of this infection‐generated bone loss. PRRs represent an important target to explore this issue as the first defence molecules that mediate host recognition of invading infectious pathogens. NOD1, a representative PRR from the NLRs family, has been reported to interfere with periodontitis by triggering immune responses.^22^ However, its function in osteogenic differentiation remains unknown. Our study established a direct connection between NOD1 activation and periodontal bone homeostasis for the first time.

The expression of NOD1 in the oral cavity has been proven by various research including oral epithelial cells, pulp fibroblasts, gingival fibroblasts, and periodontal ligament cells.^31^, ^32^, ^33^, ^34^ Okugawa et al. exposed NOD1‐expressing human embryonic kidney cells to heat‐killed periodontitis‐related bacterial and proved that NOD1 recognizes fragments from periodontal pathogens.^35^ NOD1 is more prevalent than other NLRs in healthy periodontal tissues. Moreover, *P.g*, *A.a*, and *F.n* infection may result in a high level of NOD1 expression in periodontium.^22^, ^24^, ^26^, ^27^ In our study, we tested the NOD1 protein level and the intensity of IHC and IF in periodontal tissues collected from healthy people and patients with periodontitis. We observed that NOD1 is substantially expressed in periodontal tissue in individuals with chronic periodontitis. The elevated NOD1 expression suggests NOD1 might be an important receptor for the recognition of periodontal pathogens in periodontitis.

On the basis of NOD1 indisputable high expression in bacterial‐infected periodontal tissue, the influence of NOD1 regarding alveolar bone homeostasis needs to be expounded. HPDLSCs are one of the major cell sources with the osteogenic potential to form alveolar bone.^36^ Autologous or allogeneic PDLSCs treatment shows successful regeneration of periodontal soft and hard tissues in the animal model of periodontitis.^37^, ^38^ Moreover, periodontitis‐related PAMPs such as lipopolysaccharide (LPS) have been proved to jeopardize the osteogenic capacity of hPDLSCs.^6^, ^7^ IE‐DAP from PGN structure is an important PAMP recognized by NOD1, its synthesis is significantly increased in periodontitis patients' subgingival plaque.^10^ The mice periodontitis model has shown that PGN and LPS can synergistically enhance bone resorption and osteoclastogenesis.^39^ It is crucial to verify whether stimulation from PGN containing iE‐DAP can influence the osteogenic potential of hPDLSCs via NOD1.

In our study, we firstly confirmed that with DAP treatment, NOD1 in hPDLSCs can be activated. We then tested the osteogenic capacity of hPDLSCs under DAP treatment and surprisingly found out that with NOD1 activation, the osteogenic capacity of hPDLSCs was greatly impaired. After being co‐incubated with ML130, the NOD1 receptor in hPDLSCs has been inhibited, and hPDLSCs restored the ability of osteogenesis. Similar results were found on other NLRs in different oral tissue. Up‐regulation of NOD2 significantly suppressed odontoblast differentiation of human dental pulp cells.^40^ As far as we know, this research is the first indication that the DAP‐induced NOD1 activation is directly implicated in the osteogenic process in hPDLSCs.

In oral epithelial cells and gingiva fibroblasts, microbe‐induced NOD1 activation and subsequent inflammatory responses are mediated by activation of the MAPK pathway.^31^, ^32^ In hPDLCs, tri‐DAP led to the increased production of pro‐inflammatory cytokines via activation of MAPKs.^27^ Previous studies also have shown that osteoblastic cell differentiation can be regulated by MAPK pathways.^41^, ^42^ Therefore, we started to focus on the MAPK pathway to check if they are involved in this osteogenic inhibition process of DAP‐NOD1 complex. With DAP stimulation in hPDLSCs, we noticed both time and dose‐dependent phosphorylation of p38. The addition of ML130, a NOD1‐specific inhibitor, reduced these elevations in p38 phosphorylation, confirming that NOD1 activation can stimulate p38 downstream signalling in hPDLSCs. We also examined the classical pathways regulating osteogenesis such as the Wnt/β‐catenin pathway. However, such dose‐dependent activation was not seen with respect to total β‐catenin and active β‐catenin under DAP treatment (1–10 μg/ml) (Figure S4). To further expound the involvement of p38 MAPK in the NOD1‐regulated osteogenesis in hPDLSCs, we managed to abolish the DAP‐induced increase of phosphorylated p38 using the p38 inhibitor, SB203580. And these biochemical alterations in p38 also coincide with the osteogenic differentiation phenotype we observed previously. Blocking p38 phosphorylation partially restored the DAP‐impaired osteogenic capacity of hPDLSCs. Corresponding evidence from the literature has demonstrated that p38 MAPK appeared to be crucial for the differentiation of osteoblastic cells.^43^, ^44^

**FIGURE 6:**
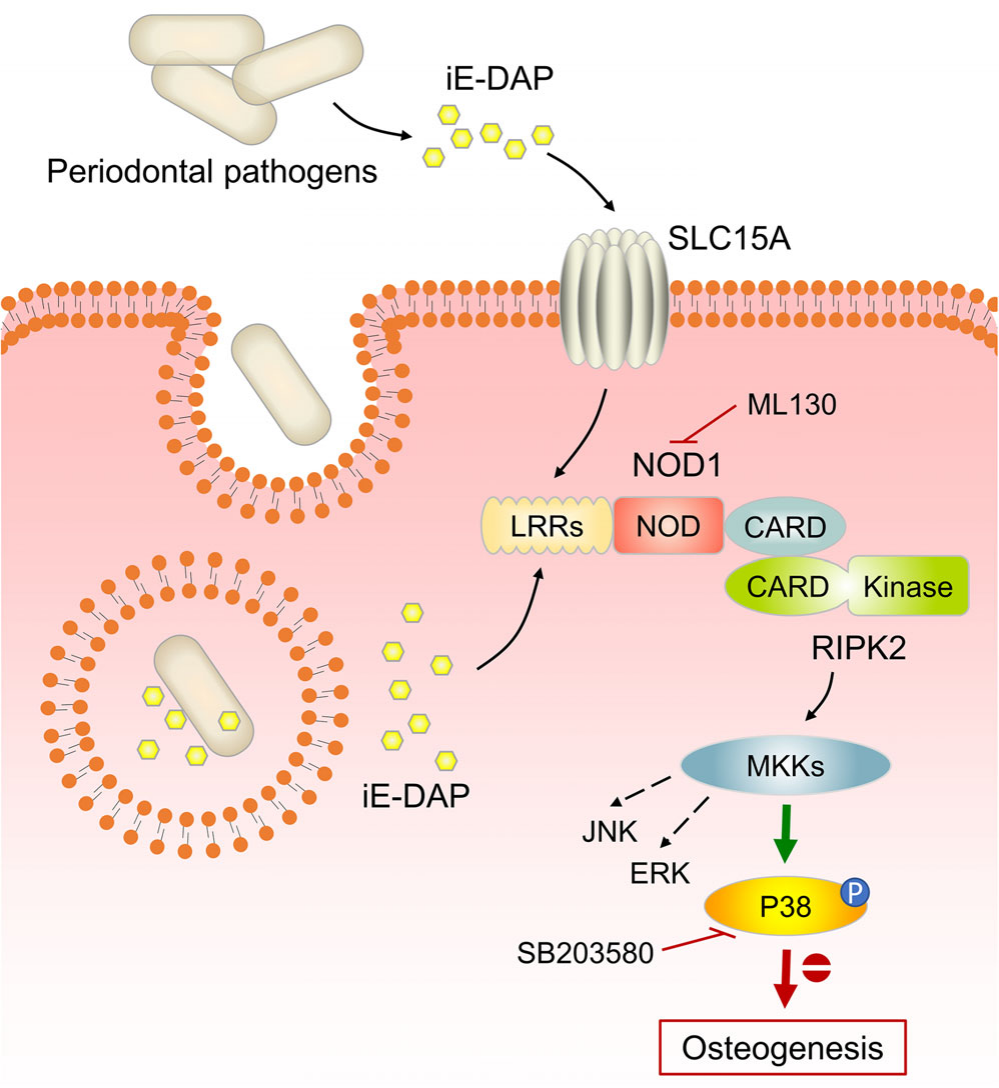
The schematic figure illustrating the biomechanics of NOD1‐regulated osteogenesis in hPDLSCs. IE‐DAP enters cells through members of the solute carrier family 15 (SLC15A) or internalization by phagocytosis. Then, iE‐DAP activated the cytosolic NOD1 receptor and induced the p38/MAPK signalling pathway, which negatively regulated the osteogenic and differentiation of hPDLSCs.

In conclusion, this study is the first to explore the role of the NOD1 receptor, an important innate immune PRR, in the osteogenic differentiation of hPDLSCs (Figure 6). We have identified NOD1 receptor as a novel target in oral microbe‐mediated alveolar bone loss. The high expression of NOD1 in chronic periodontitis was confirmed in our study, and NOD1 activation by DAP reduced the osteogenic potential of hPDLSCs. We managed to restore the NOD1‐impaired osteogenesis at two levels: at the NOD1 receptor level utilizing ML130 and at the main signalling molecule p38 level using SB203580. These data strongly confirm that NOD1 is an important receptor mediating the interaction between oral microbe and hPDLSCs. This might provide a potential target for the treatment of periodontitis and alveolar bone repair.

## AUTHOR CONTRIBUTIONS

Yuying He, Chenchen Zhou, and Shujuan Zou designed the study topic and experiments. Yuying He, Zuping Wu, Sirui Chen, Jiahe Wang, and Li Zhu performed the experiments. Yuying He and Chenchen Zhou accomplished data analysis and prepared the manuscript. Jing Xie and Shujuan Zou evaluated the manuscript and data reliability. The article was reviewed by all writers. Shujuan Zou granted final consent to the published version.

## CONFLICT OF INTEREST

The authors report no conflicts of interest.

## Supporting information


**Figure S1** Identification of hPDLSCs extracted from periodontal ligaments.

**Figure S2** Periodontal ligament tissue samples collection.

**Figure S3** The cytotoxicity of Tri‐DAP on hPDLSCs.

**Figure S4** Western blotting results of Wnt/β‐catenin pathway.

**Table S1** Primer sequences of control gene and osteogenesis‐related genes in hPDLSCs for RT‐qPCR.

## Data Availability

The present research makes no reference to publicly accessible or shareable data. All original data that support the conclusions of this work are available from the corresponding author upon reasonable request.
