# Supplementary figures and images for: Activation of the pattern recognition receptor NOD1 in periodontitis impairs the osteogenic capacity of human periodontal ligament stem cells via p38/MAPK signalling
Source: Cell Prolif. 2022 Aug 31;55(12):e13330. doi: 10.1111/cpr.13330 (PMC9715354; doi:10.1111/cpr.13330)

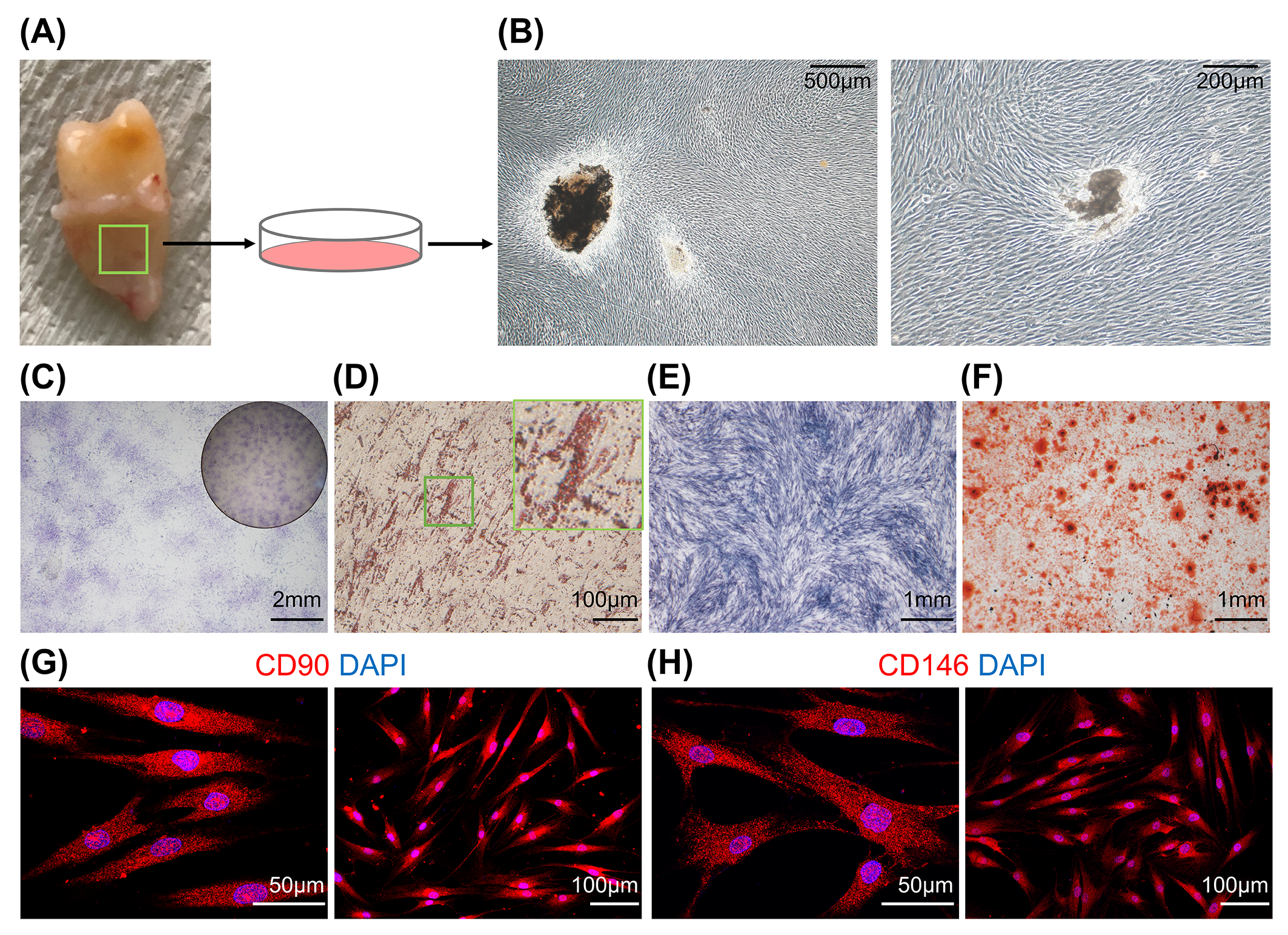

Supplement: Supplementary file 1 — Figure S1 Identification of hPDLSCs extracted from periodontal ligaments. [file CPR-55-e13330-s002.tif]

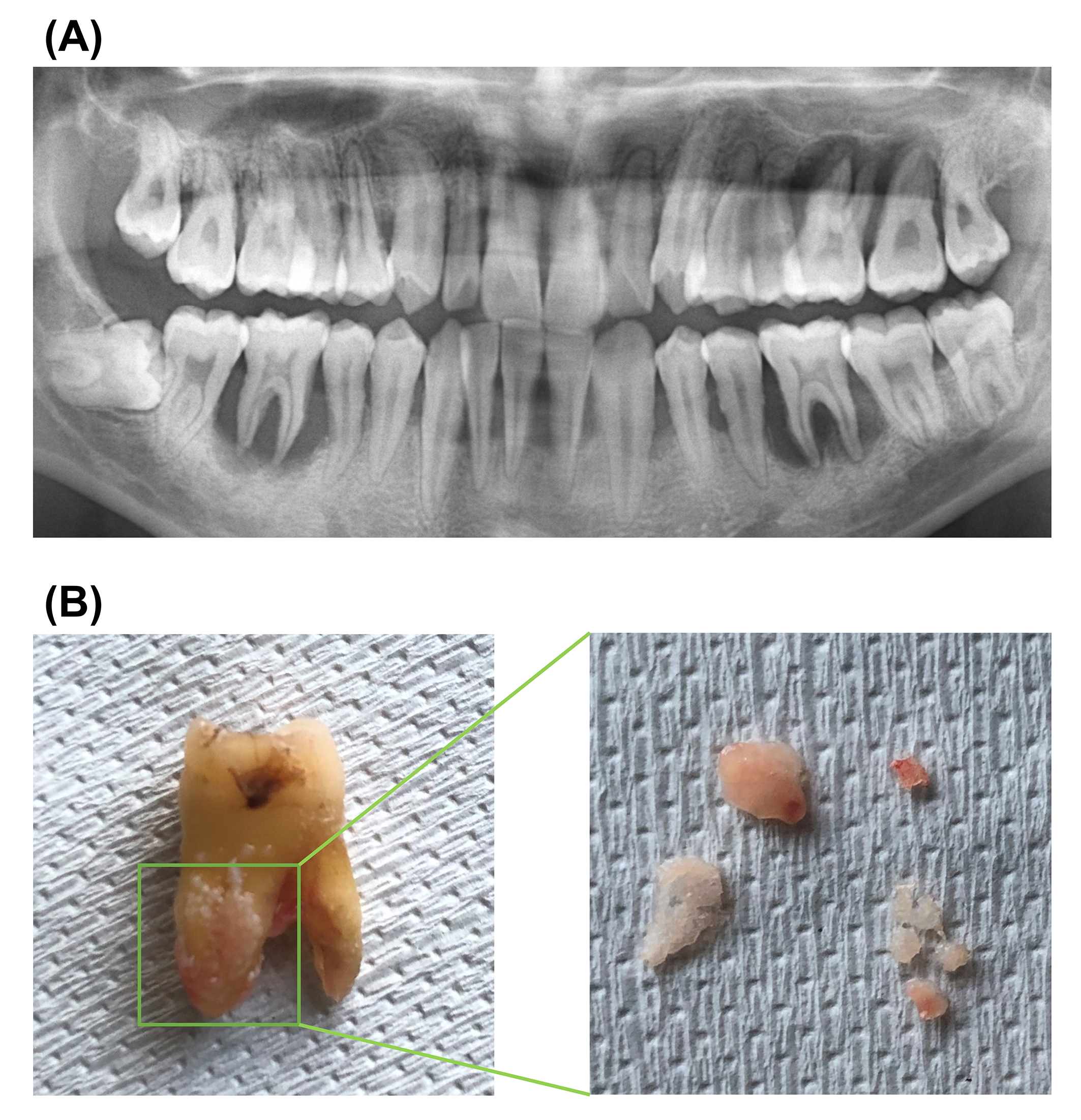

Supplement: Supplementary file 2 — Figure S2 Periodontal ligament tissue samples collection. [file CPR-55-e13330-s003.tif]

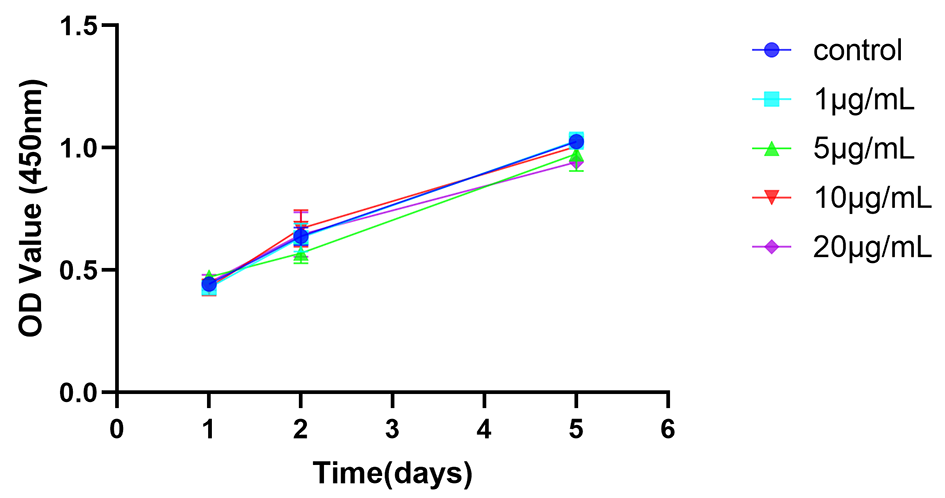

Supplement: Supplementary file 3 — Figure S3 The cytotoxicity of Tri‐DAP on hPDLSCs. [file CPR-55-e13330-s001.tif]

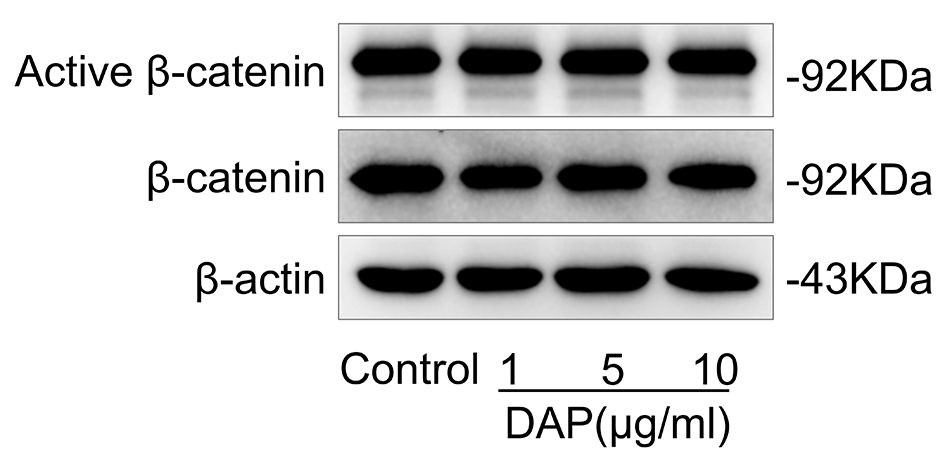

Supplement: Supplementary file 4 — Figure S4 Western blotting results of Wnt/β‐catenin pathway. [file CPR-55-e13330-s005.tif]
