# Supplementary material for: Activation of the pattern recognition receptor NOD1 in periodontitis impairs the osteogenic capacity of human periodontal ligament stem cells via p38/MAPK signalling
Source: Cell Prolif. 2022 Aug 31;55(12):e13330. doi: 10.1111/cpr.13330 (PMC9715354; doi:10.1111/cpr.13330)
Supplement: Supplementary file 5 — Table S1 Primer sequences of control gene and osteogenesis‐related genes in hPDLSCs for RT‐qPCR. [file CPR-55-e13330-s004.docx]

**Supplemental Table 1. Primer sequences of control gene and osteogenesis-related genes in hPDLSCs for RT-qPCR**

| Gene name | Gene ID | Primer pairs |
| --- | --- | --- |
| GAPDH | NM_001256799.3 | Forward: GACAGTCAGCCGCATCTTCT  Reverse: GCGCCCAATACGACCAAATC |
| SP7 | NM_001300837.2 | Forward: CTCCTGCGACTGCCCTA  Reverse: GCGAAGCCTTGCCATACA |
| COL1A1 | NM_000088.4 | Forward: CCCCGAGGCTCTGAAGGT  Reverse: CACCAGCAATACCAGGAGCA |
| RUNX2 | NM_001015051.4 | Forward: TCGCCTCACAAACAACCACA  Reverse: CTGGTAGTGACCTGCGGAGA |
